# AI and big data driven knowledge mapping of exosome–hydrogel research in orthopedic regeneration and tissue engineering

**DOI:** 10.3389/fcell.2026.1786225

**Published:** 2026-02-17

**Authors:** Qinghan Li, Liming Lou, Shuaishuai Wang, Minglei Zhang

**Affiliations:** 1 Department of Xinmin Orthopedic, China-Japan Union Hospital of Jilin University, Changchun, China; 2 School of Technology, Beijing Forestry University, Beijing, China

**Keywords:** artificial intelligence, big data analytics, bone regeneration and tissue engineering, exosome–hydrogel systems, knowledge graph

## Abstract

**Background:**

Exosome-hydrogel complexes have great potential in regenerative medicine, being able to combine biological signals with structural support. But overall, the knowledge structure and translational connections between academic discoveries and patent deployment are not clear.

**Methods:**

A dual-source analysis framework was established to analyze academic papers and patents, illustrating the landscape of exosome–hydrogel research from 2016 to 2025. An interdisciplinary knowledge graph was constructed using topic modeling, entity–relation extraction, and evidence-ranking methods to quantify temporal trends, thematic differences, and translational gaps.

**Results:**

The core components include mesenchymal stem cell–derived exosomes and hydrogels based on gelatin methacrylate (GelMA) or collagen, which form a well-established research foundation. Academic research focuses on osteogenesis, and recent progress mentions angiogenesis and immune regulation. The research application has strong temperature dependence, and patent activities lag behind academic publications. Several high-evidence yet unpatented propositions, such as “hydrogel-encapsulated exosomes” and “exosome-enhanced angiogenesis,” represent potential innovation opportunities.

**Conclusion:**

This study employs a data-driven framework to connect scientific research with transformation. The integration of semantic models and cross - source evidence reflects the evaluation logic of exosome - hydrogel research, and provides support for future research in the field of regenerative biomaterials and the priority of patent strategies.

## 1 Introduction

Tissue regeneration and repair are the core of regenerative medicine. The key challenge lies in the multi-dimensional coupling of cell regeneration, signal transduction, and structural support. Extracellular vesicles, especially exosomes, are key mediators of intercellular communication. Exosomes contain active molecules such as proteins, lipids, and nucleic acids, and can promote cell proliferation, angiogenesis, and tissue regeneration (Li et al., 2018; Chen et al., 2019; Luo et al., 2019; Zhang et al., 2022).

Hydrogels have adjustable mechanical properties, biocompatibility, and the characteristics of 3D scaffolds. They can simulate the natural extracellular matrix and provide an ideal microenvironment for cell adhesion, differentiation, and tissue repair (Lu et al., 2024; Kim et al., 2022). In recent years, the exosome-hydrogel composite system is a promising strategy in regenerative medicine, especially in bone defect repair (Chen et al., 2023; Wu et al., 2024; Yang et al., 2023; Li et al., 2024a; 2024b; Lu et al., 2023). His hybrid system can enable exosome release or delivery, and regulate cell behavior or microenvironment through the hydrogel network, providing a stable alternative for traditional cell therapy (Zhao et al., 2022; Isik et al., 2023; Sun et al., 2023).

Numerous related studies have been reported. However, current studies on the exosome–hydrogel system still have several deficiencies. Most existing studies focus on specific experimental models or application scenarios. For example, studying the effects of exosomes from different sources on osteogenesis, angiogenesis, and immune microenvironment regulation (Fan et al., 2020; Chen et al., 2025; Liu et al., 2023), different hydrogel materials were evaluated from the aspects of encapsulation efficiency, injectability, and biocompatibility. However, the research lacks systematic integration and it is difficult to form a comprehensive structured knowledge system.

In the context of the rapid expansion of research achievements, it becomes important to understand the connection between academia and patents. The patent system can reflect the technical maturity and industrial interest. Even so, there are few systematic analyses on academic-patent transformation, especially the analysis using repeatable frameworks for text mining or evidence linking (Tseng et al., 2007; Yoon and Park, 2004; Du et al., 2020; Zhang et al., 2017). There was still no framework for quantitative cognitive to bridge the basic discoveries and translational applications, and there was no overall outline of academic hotspots, technological gaps, and innovation paths. Unlike previous narrative reviews, this study quantitatively links scientific literature and patent data through semantic modeling, providing a reproducible computational framework for translational biomaterials research.

It is important to develop a cross - source and systematic method to quantitatively figure out the knowledge architecture and transformation mode of the exosome-hydrogel system. The traditional one is a narrative review, while this study uses large-scale text mining and semantic modeling, goes beyond single research results, and integrates scientific literature and patent data into a unified analysis framework to reconstruct the macro - research appearance (Lee et al., 2020).

A semantic analysis and knowledge extraction framework is proposed based on corpus (academic papers and patents). This framework combines topic modeling, entity recognition, relation extraction, time series analysis, and evidence strength ranking to construct a traceable evidence chain, connecting academic discoveries with patent layouts. Three core research questions are solved: (1) What are the core topics and semantic structures of the current exosome-hydrogel research? (2) How do these topics evolve over time? (3) Which high-evidence academic propositions are not protected by patents, that is, what are the potential transformation opportunities?

This section introduces the Abstraction–Topic modeling–Data association–Strength ranking (ATDS) framework, which systematically connects. This method quantifies the time synchronization and semantic differences in the academic and patent fields, and takes into account the time lag of “paper to patent”. The research results show that academic research mainly focuses on the axis of “mesenchymal stem cells (MSC)- hydrogel-bone regeneration”, and patent behavior presents the characteristic of institution expansion in the claim language, and there is a semantic gradient of “research focus to application expansion”.

Further entity and relationship analysis shows that the exosome source, hydrogel matrix, and functional endpoints have evolved over 10 years, depicting a comprehensive knowledge framework of “biological input-matrix carrier-functional output”. This framework provides quantifiable insights and a batch of candidate proposals for subsequent research and transformation and development.

This study interprets the exosome-hydrogel system from three aspects: semantics, entities, and relationships, and reveals the internal knowledge logic from basic discoveries to patent transformation. It fills the gap in the key aspects of regenerative biomaterial research, and also introduces a reusable computational framework through data-driven evidence, which can connect academic research with industrial applications. This method has three key contributions: (1) helps researchers identify academic hotspots and the trends of research evolution; (2) provides decision-making support for patent strategies and technology planning; (3) constructs an interpretable and traceable knowledge graph model, promoting the structured development and practical transformation of regenerative biomaterial research.

## 2 Materials and methods

**FIGURE 1:**
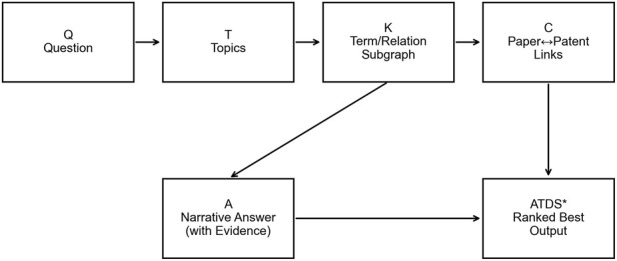
Conceptual workflow of the ATDS framework.

This study adopts a framework that integrates evidence (literature and patents), a terminology/relationship-based knowledge graph, and a way of evidence strength ranking. It systematically characterizes the knowledge structure, hot spot evolution, and transformation opportunities of exosome/vesicle-loaded hydrogels for bone repair. The overall analysis work flow is as follows:

The overall work flow is from Q to T to K to C to A, as shown in Figure 1, which contains the organization of evidence chains for result generation and the display of logical processes.

### 2.1 Data collection and corpus construction

This study uses the academic literature and patent document corpus system to process structured text data across knowledge domains. Its purpose is to systematically display the research evolution and technical transformation trajectory of the exosome-hydrogel system in the field of bone regeneration. The data covers from 2016 to 2025, integrating scientific research achievements (in academic aspects) and industrial technology layout (in patent aspects), and realizing cross-source semantic linking between “research” and “application”.

Academic papers were retrieved from major international journal databases based on titles, abstracts, or keywords containing the core terms “exosome/extracellular vesicle,” “hydrogel,” and “bone regeneration/bone repair.” Patent data were collected from open intellectual property databases using identical filtering criteria. After importation, all documents were uniformly formatted to ensure consistency across key fields, including unique identifier, title, abstract, and year of publication or disclosure.

To ensure the consistency and comparability of time, convert the year field to an integer and unify to the same time series. Academic data mainly shows the research focus and mechanical verification, while patent data reflects the scope of the claims of applied technology and the formalization of language. he two corpora jointly provide a panoramic “discovery-verification-translation” knowledge framework (Porter and Cunningham, 2004).

### 2.2 Text preprocessing and topic modeling

Before topic modeling and before entity analysis, the corpus needs to undergo systematic preprocessing and quality control. Concatenate the titles and abstracts of the documents so that the context can be more coherent. Delete texts with a length of less than 30 characters, which can reduce semantic noise and screen out ambiguous samples. Replace missing values or null values with empty strings to prevent the model from interrupting.

To ensure consistency of vocabulary at the language level the text is lowercased and punctuation is removed stopwords are filtered using standard english lists and domain specific word lists with scientific terms such as “gelatin methacryloyl (GelMA)”, “MSC”, “osteogenesis” retained the standardization process follows traditional natural language processing pipelines such as tokenization stopword management and text normalization (Loper and Bird, 2002).

In order to avoid deviation in time analysis, the annual statistical data will adopt the full year (up to 2025) to prevent false decline due to incomplete data. Then the text is constructed into a standard and quality-controlled corpus for semantic models and entity analysis.

To find the differences in semantic structures between academic texts and patent texts, this paper uses latent Dirichlet allocation (LDA) for topic modeling. The text is vectorized using a bag-of-words model that includes co-occurrences of unigrams and bigrams to enhance the recognition ability of compound terms. To avoid the dilution of topics caused by terms being too frequent or too rare, an upper threshold (≤90% document frequency) and a lower threshold (≥2 occurrences) are set to focus on semantically useful terms. The number of topics was determined based on coherence score and corpus size to ensure semantic stability.

The LDA model produced a document–topic distribution matrix assigning a set of topic weights to each record. The number of topics was adapted to corpus size: 10 topics for >200 documents, eight topics for 80–200 documents, and 5 topics for <80 documents. Each topic included the top 12 weighted keywords for interpretability. Model training used symmetric Dirichlet priors (α = 0.1, β = 0.01) to enhance sparsity and interpretability, consistent with classical applications of LDA in scientific text mining.

To realize the visual semantic pattern, the document-topic matrix is projected into a two-dimensional space by using the method of truncated singular value decomposition (SVD). The first and second principal components (dimension 1, dimension 2) are used as the main and secondary semantic axes, in the same way as latent semantic analysis (Deerwester et al., 1990).

### 2.3 Entity and relation extraction

After semantic modeling, entity and relationship extraction form a structured knowledge graph in this field. Using NLP technology to identify/standardize entities, and processing hydrogel pads, exosome-derived cells, and experimental endpoints/biomarkers. Standardize synonyms and names to simplify and remove lexical fragments, for example, merge “Gelatin methacrylate” and “GelMA”, and merge “microcomputed tomography” and “micro-CT.” This is in line with the already constructed biomedical NLP framework (Neumann et al., 2019).

Integrate the extracted entities into annual series to grasp long-term trends. Among hydrogels, GelMA, collagen, chitosan, hyaluronic acid (HA), and polyethylene glycol are the main materials. MSC-derived exosomes still rank first among source cells, and bone marrow–derived mesenchymal stem cell (BMSC), adipose-derived stem cell (ADSC), and immune cells are gradually increasing. In terms of end-point indicators, osteogenic markers (ALP, RUNX2, OCN) and angiogenesis markers (VEGF, CD31) are the most common.

Extracting relations uses a mixed method, combining dependency parsing and lexical-syntactic pattern matching. Generating verb-centered relation triples, with document types (paper and patent) and years, to assist downstream hypothesis identification and evidence quantification. Dependency parsing and relation extraction follow the Stanford natural language processing toolchain, such as word segmentation, part-of-speech tagging, syntactic dependency parsing (Manning et al., 2014). This transforms narrative statements into computable knowledge structures and descriptive evidence into structured data. Typical dependency-based relation extraction patterns were used to identify triplets such as “hydrogel–promotes–osteogenesis” and “exosome–enhances–angiogenesis,” enabling conversion of descriptive statements into computable knowledge structures.

### 2.4 Analytical framework and visualization

Knowledge extraction after semantic modeling, and analyzing the macro-evolution of papers and patents.

The annual number of paper publications and the number of patent disclosures form approximately parallel time-series curves. The form and synchronization of the curves reflect the growth rate of research and application, and can evaluate the time-coupling relationship between scientific exploration and technological deployment.

The frequency of key entities in the annual period is tracked by entity-level analysis, and trend lines are drawn for the top 12 high-frequency elements within the categories of matrices, cellular sources, and endpoint categories. These curves show the changes of research focuses over time.

Build a matrix to grasp the pattern of experimental configurations. Rows are hydrogel pads, columns are functional endpoints, and cells are co-occurrence frequencies. Heatmaps visualize high-density clusters as experimental paradigms, and sparse areas are areas of experiments or emerging research, which is the same as the co-word analysis classification of scientific knowledge graphs (Van Eck and Waltman, 2010).

### 2.5 Evidence ranking and interpretation

Finding innovative directions with strong academic evidence but low industry adoption requires cross-field comparison at three levels. Sort the relationships not appearing in patents in academic literature according to the citation frequency to obtain the attribute of high evidence and unpatented. Typical examples are “hydrogel-encapsulated exosomes,” “GelMA-enhanced osteogenesis,” “exosome-enhanced angiogenesis.” The differences between academic knowledge and patent claims have long been studied in scientometrics and patentology.

Decompose the results, and decompose into mature themes and emerging themes. For each relationship, calculate and compare the total number of evidences and the number of evidences in the past 3 years. The relationship where both dimensions are at a high level is a sustained hot spot (stable trajectory), and the relationship where the total number of evidences is at a medium level but the recent growth is relatively fast is an emerging frontier. Measure the priority of research focus through two-dimensional sorting, and divide into established consensus areas and new innovative frontiers.

This framework transforms traditional qualitative research into quantitative analysis of knowledge prioritization, and also identifies innovation nodes of “science and mathematics fields but insufficient institutional support.” It provides data-driven guidance for future research directions, experimental verification patent strategies. Finally, this study constructs a traceable workflow, including corpus modeling, relationship identification and evidence ranking, and realizes the closed-loop connection between academic inquiry and technological transformation (Jaffe, 2002). This work is entirely data-driven, integrating information from academic publications and patent records.

## 3 Results

### 3.1 Annual evolution of publications and patents

**FIGURE 2:**
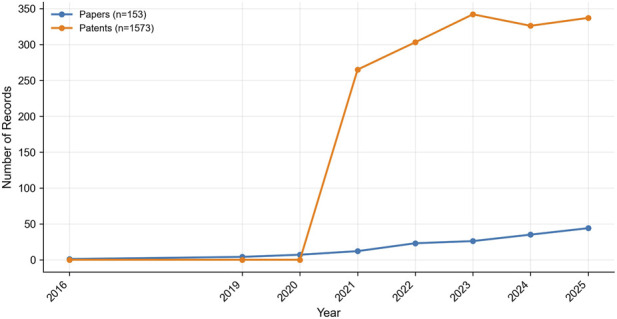
Annual evolution of publications and patents in exosome–hydrogel research from 2016 to 2025.

To demonstrate the research achievements and technological transformation dynamics of exosome-hydrogel systems in the past decade, an annual aggregation analysis of the literature and patents published from 2016 to 2025 is carried out (Figure 2).

The result shows a “bimodal pattern,” with stable academic growth coexisting with concentrated patent expansion, reflecting a phased shift from theoretical accumulation to application transformation. In terms of academia, since 2016, the number of papers has steadily increased, growing from early exploratory research to more than 40 papers in 2025, showing a continuous upward trend. This continuous growth indicates that the understanding of the exosome-hydrogel system in regenerative medicine in the field of scientific communication is continuously deepening, marking the shift from conceptual exploration to mechanism elucidation and experimental verification.

Early research focused on the isolation, purification, and physical and chemical analysis of exosomes. After 2019, research shifted to carrying out work related to vector construction and functional verification. For example, exosomes from mesenchymal stem cells were applied in hydrogels for bone regeneration. After 2022, matters related to exosomes in interdisciplinary areas such as immunomodulation-tissue repair began to emerge, which indicates that the research scope has expanded in the direction of multi-mechanism integration.

In 2021, there was a significant increase in industrial patent output, which increased by approximately two times in 3 years and remained at a high level from 2023 to 2025. The rapid growth reflects the maturity of technology and the increase in industrial investment. The time of some patents and academic achievements is consistent, which indicates that there is a mode of co-evolutionary research - patent between research institutions and enterprises. This synchronism is relatively rare in the field of biomaterial research, which shows that the exosome - hydrogel system is feasible and its market potential is widely recognized.

The time trajectories of papers and patents are positively correlated. Continuous academic output lays the foundation for technology transfer, and active patent applications promote the in-depth development of research focuses. Essentially, a two-way coupling between academia and industry has been formed: academia takes the lead in mechanical discoveries and method innovations, and industry is in a leading position in technical standards and intellectual property protection. This cyclic process “scientific exploration → engineering verification → industrial application” is the driving force behind the research progress of exosome hydrogels.

### 3.2 Topic structure and semantic divergence between papers and patents

**FIGURE 3:**
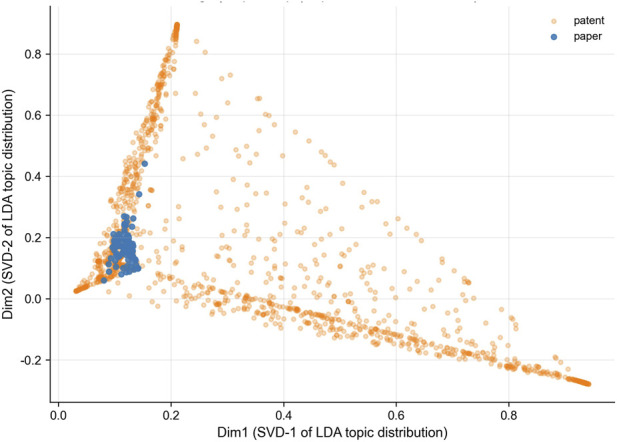
Semantic structure and topic divergence between academic papers and patents. Visualization based on Truncated SVD projection of LDA topic distribution.

Using LDA topic model and truncated SVD dimensionality reduction visualization to compare academic resources and patent layouts in the merged corpus (Figure 3). This method can intuitively explain the differences in narrative patterns, semantic concentration, and technical diversity between the two fields.

After dimensionality reduction, it is observed that academic samples are relatively closely clustered within the semantic space, while patents are in a dispersed state. This difference reflects that there are differences in narrative logic: scholars, based on mechanism, attach importance to causal chains and experimental verification; patents use non-standard claim terms to carry out extensive technical coverage. Academic texts have high semantic density and focus on content, while patents have a relatively wide semantic range and strong universality.

Further topic statistics indicate systematic divergence in research emphasis (Table 1). On the academic side, Topic 2 and Topic 3 dominate, with mean topic weights of 0.3870 and 0.4935, respectively—significantly higher than 0.0720 and 0.0804 on the patent side. Keywords for these topics include “MSC,” “hydrogel,” “osteogenesis,” “bone regeneration,” “scaffold,” and “exosome,” forming the core narrative of academic research. This structure indicates that academic discourse is semantically convergent, focusing on the mechanistic core of exosome–hydrogel systems in bone regeneration, reflecting a mature research consensus.

**TABLE 1:** Topic modeling summary of the exosome–hydrogel corpus (2016–2025).

Topic	Short label	Representative keywords	Primary source	Mean topic weight (paper)	Mean topic weight (patent)
1	Exosome characterization and methods	Evs, vesicles, extracellular, tissue, derived, method	Patent	0.0331	0.0466
2	MSC-derived exosome–hydrogel composites	Cells, stem, bone, mesenchymal, exosomes, regeneration, hydrogel	Mixed (paper-dominant)	0.3870	0.0720
3	Exosome–GelMA system for osteogenic repair	Bone, hydrogel, exosomes, regeneration, scaffold, osteogenic	Mixed (paper-dominant)	0.4935	0.0804
4	Therapeutic and delivery claims	Methods, compositions, delivery, therapeutic, systems	Patent	0.0121	0.1020
5	Material composition and surface modification	Bone, cell, material, exosomes, surface, nitride	Patent	0.0533	0.0721
6	Compound- and RNA-related inventions	Composition, invention, compound, acid, rna	Patent	0.0019	0.0736
7	Surgical device and process systems	Surgical, device, ultrasonic, control, disclosure	Patent	0.0039	0.0929
8	Broad therapeutic applications	Cells, compositions, treating, cancer, disease, use	Patent	0.0007	0.2564
9	Gene and molecular expression regulation	Gene, dna, expression, rna, disease	Patent	0.0069	0.0342
10	Composite systems and multifunctional use	Engineered, nucleic, compositions, systems, embodiments	Patent	0.0077	0.1698

In contrast, the patent domain shows a dispersed thematic structure. Topics 4, 6, 7, 8, 9, and 10 are patent-dominant, featuring keywords such as “treatment,” “composition,” “system,” “method,” “device,” and “embodiment”—all characteristic of claim-oriented expressions. This pattern reflects the horizontal expansion strategy of patents, which prioritize breadth of coverage and claim protection over mechanistic specificity. Frequent patent keywords such as “broad therapeutic application,” “system and process design,” and “composition formulation” exemplify this institutionalized generalization.

In conclusion, academic research is in a state of vertical deepening (focusing on refinement), and patents are in a state of horizontal expansion (broadening the concept boundaries). The two are not in an opposing relationship but complementary stages of knowledge transformation: academic research endows mechanisms with depth, and patents transform mechanisms into technically feasible forms that can be popularized and protected.

### 3.3 Temporal evolution of materials and cell sources

**FIGURE 4:**
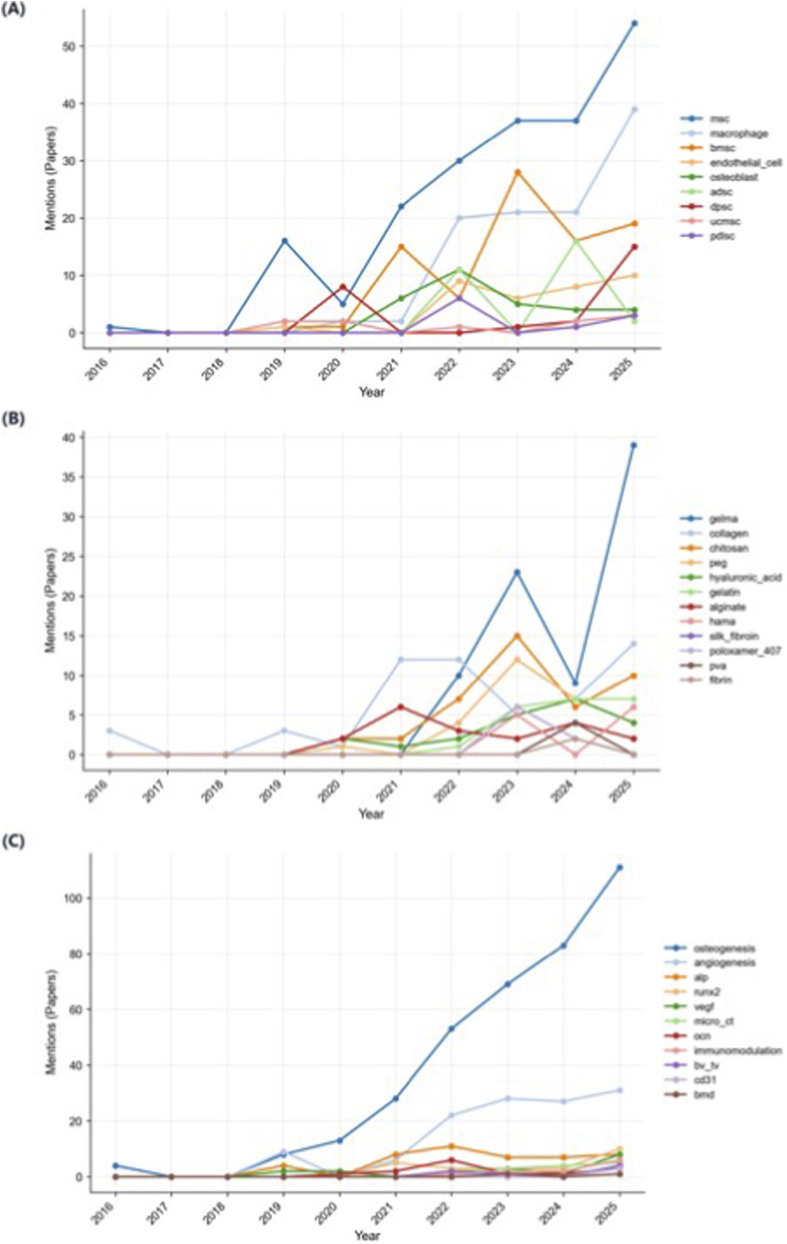
Temporal evolution of entities related to exosome–hydrogel research (2016–2025). **(A)** exosome source cells, **(B)** hydrogel materials, and **(C)** outcome and biomarker indicators.

To clarify the long-term evolution of the exosome-hydrogel system, the annual trajectories of key research entities are analyzed from three dimensions: exosome-derived cells, hydrogel matrices, and evaluation endpoints or biomarkers (Figure 4). They jointly constitute a three-layer framework of biological input, matrix carrier, and functional output.

#### 3.3.1 Evolution of exosome source cells

At the cellular level (Figure 4A), the frequency of positive osteoblasts of MSCs reached its peak during 2025. Mesenchymal stem cell exosomes have the characteristics of stable source, low immunogenicity and strong biological activity, and are standardized biological inputs in the research of bone regeneration and tissue repair. In the past decade, evidence has been continuously accumulated from “isolation and characterization” to “loading and delivery”, and more and more attention has been paid to using hydrogel carriers to achieve target-related and microenvironment regulation.

In recent years, exosomes have various sources. Since 2022, macrophage exosomes have developed rapidly, and BMSC-derived, ADSC-derived, periodontal ligament stem cell -derived, and immune cell exosomes have also been mentioned more. This diversity indicates that the research paradigm has shifted from a single MSC framework to a multi - source system, emphasizing immune regulation and tissue-specific functions. The new paradigm transforms the super-structural bone repair into immune-regeneration coordination, regarding exosomes as dynamic regulators of the microenvironment rather than just passive delivery vehicles.

#### 3.3.2 Evolution of hydrogel materials

Hydrogel materials are the core of exosome carriers, directly related to system control and biological response. Figure 4B analysis shows that GelMA and collagen have become two major research branches, both showing a significant upward trend after 2020 and reaching a peak in 2025. GelMA has adjustable cross-linking density, mechanical strength, and good biocompatibility, and has become the first choice for the construction of biodegradable scaffolds. Collagen has the characteristics of natural materials, excellent cell adhesion ability, and good performance in simulating the physiological microenvironment. These materials coexist, showing a balance between engineering control and biomimetics.

Chitosan, polyethylene glycol, HA and alginate have periodic fluctuations. Explore how physicochemical changes affect exosome stability and release kinetics. For example, polyethylene glycol and chitosan can enhance mechanical properties and release performance. HA and alginate regulate cell adhesion, porosity and degradation rate. The material system shifts from dispersion exploration to core-temperature with peripheral innovative modification. Gelatin methacryloyl and collagen provide stable and functional materials, promoting the expansion of performance and mechanical properties.

#### 3.3.3 Evolution of evaluation endpoints and biomarkers

Under the functional level (Figure 4C), osteogenesis is the core evaluation endpoint, which has grown rapidly since 2020 and reached a peak in 2025. The core osteogenesis markers, such as ALP, RUNX2, OCN, micro-CT, BV and TV, are reported the most, demonstrating that osteogenesis is in a dominant position in the main research axis.

Since 2021, the levels of vascular-related markers have increased rapidly, with VEGF and CD31 playing a dominant role, indicating the existence of bone-vascular coupling. This means that the research has shifted from bone repair to integrated tissue reconstruction. Immune regulatory markers such as tumor necrosis factor-α, interleukin-10, and CD206 have also increased significantly, highlighting the importance of the immune-regeneration interface. This field is developing from a single direction to a multi-directional direction, forming a synergistic evaluation system, reflecting the trend of regenerative medicine evolving towards systemic functional regulation.

To further explore the relationship between materials and biological outcomes, the co-occurrence structure was analyzed.

### 3.4 Material–outcome Co-occurrence structure

**FIGURE 5:**
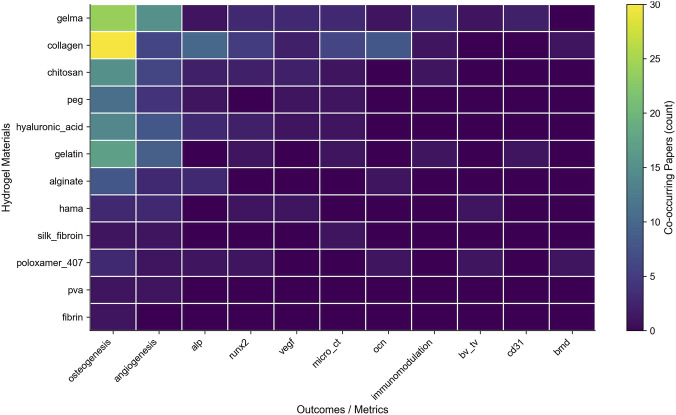
Material–outcome co-occurrence structure in exosome–hydrogel studies.

The hydrogel material appears together with biological endpoints to demonstrate the structural relationship between the material and biological results (Figure 5). The results show an obvious pattern with a higher center and lower periphery, indicating that there are some stable experimental configurations. The high - density regions match the GelMA or collagen containing osteogenic markers (ALP, RUNX2, OCN), forming a standard and repeatable model of exosome-hydrogel co-culture, which becomes the benchmark for related research.

Conversely, emerging explorations exist in the low-density peripheral region. Combinations like GelMA-VEGF, HA-CD31, chitosan-TNF-α/IL-10 have low frequencies but relatively fast development speeds, suggesting the potential to define a new wave of research paradigms. These co-occurrence patterns reveal stable experimental configurations and emerging functional relationships, which collectively provide the empirical foundation for understanding subsequent translational trends.

### 3.5 Temporal differences between papers and patents

**FIGURE 6:**
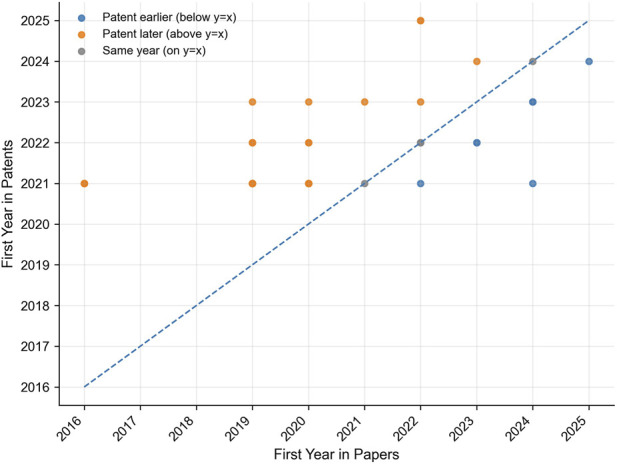
Temporal comparison of first appearance years of shared entities in papers and patents.

Building upon the material–outcome structure observed above, we next examined how these scientific findings have been translated into patented applications over time, and also displayed them with a scatter plot (Figure 6). Most data points are above the diagonal (y = x), which indicates a trend that research occurs earlier than patents. For example, classic cases such as “MSC exosome-GelMA composite material” and “exosomes promote bone repair” first appeared in academic literature around 2018–2019, but most of them obtained patents after 2021, which reflects that the expression at the institutional level is obviously lagging behind.

Points below the diagonal show the phenomenon of patent priority, especially for widely applied situations. Early applications often come from industry or academic-industry alliances, which reflects the situation of foresight and risk tolerance, meaning that the exosome-hydrogel technology is regarded as having commercial prospects before obtaining sufficient academic verification.

A small part near the diagonal points has synchronous development, which is a typical industry-university-research cooperation model. In order to realize the parallelism of paper publication and patent application, this model improves the transformation efficiency and also reflects the maturity of the organization.

Overall, a progression from unidirectional lag to multi-modal synchronization is observed, encompassing three coexisting patterns.

1. Research-led, patent-following (knowledge-driven translation)
2. Patent-led, research-validating (industry-driven foresight)
3. Synchronous co-development (integrated innovation)

These dynamics reflect a hierarchical and interactive innovative ecosystem, seeking balance between scientific depth and transformation speed, thereby enhancing the continuity from research to application in the exosome-hydrogel system.

### 3.6 High-evidence but unpatented relational propositions

**FIGURE 7:**
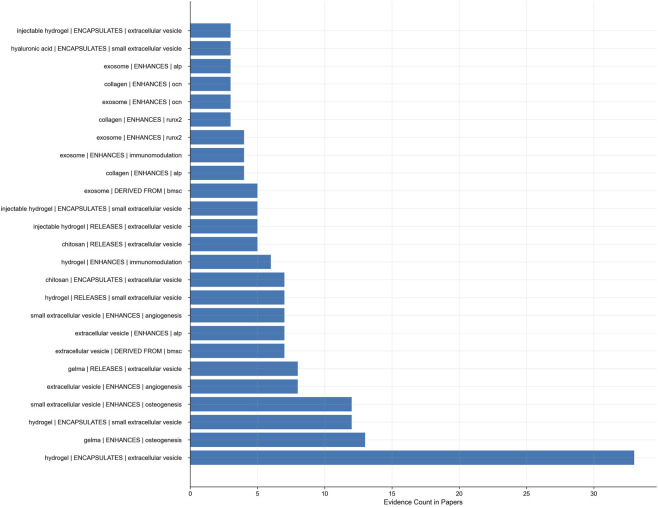
High-evidence but unpatented relational propositions extracted from paper-only datasets.

To find innovative opportunities that have been academically verified but not fully reflected in business, we compare the semantic triples (head entity, relation, tail entity) of the paper corpus and the patent corpus. Only selecting the relations that appear in highly cited academic literature, several “high-evidence unpatented” propositions are found (Figure 7).

There are groups that appear with three main mechanisms.

1. The functional association of structures, such as “hydrogel encapsulating exosomes” and “hydrogel enabling exosome release,” highlights the mechanisms of spatial distribution and related kinetics.
2. The biological effects of materials, such as “GelMA strengthens osteogenesis,” “collagen promotes the expression of ALP or OCN,” which reflecting the influence of materials on exosome activity and bone matrix formation.
3. Exosomes are associated with the microenvironment. For example, exosomes enhance angiogenesis, change macrophage polarization, and inhibit inflammation - highlighting their roles in immunity and vascular regulation.

Propositions verified in multiple studies (appearing more than 30 times on average) have reproducibility and consensus, but most are not protected by patents. The gap between evidence and protection reflects the asymmetry between academic precision and patent generality. From a strategic perspective, the unpatented relationship with high evidence support is the main goal of intellectual property development and transformation application, and it builds a bridge from scientific mechanisms to technical expressions to patent protection.

### 3.7 Identification of persistent hotspots and emerging growth points

**FIGURE 8:**
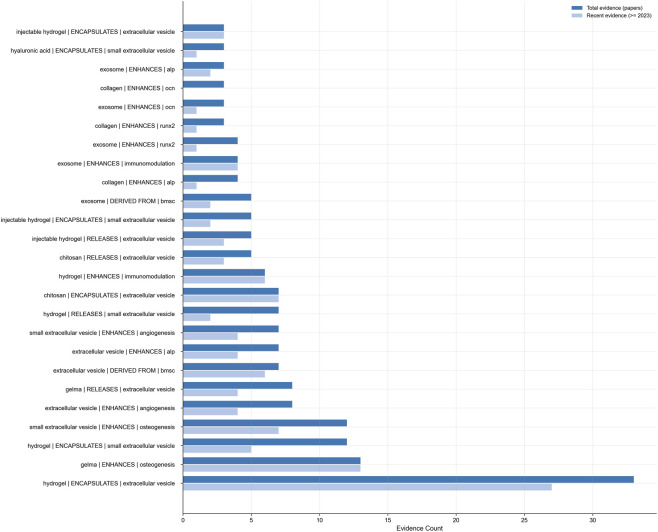
Comparison of total and recent evidence strength of candidate relations.

To distinguish between persistent hotspots and emerging growth points, a time decomposition of high - credibility, Not privatized relationships is carried out (Figure 8). By comparing the total number of evidences and the number of evidences in the recent period (2023–2025), there are two main clusters at present.

1. “GelMA enhanced osteogenesis” and “hydrogel-encapsulated exosomes” are continuous hotspots. For many years, the evidence level has been relatively high, and they are the anchors of long-term research. This reflects the standardization of the method, and it is also continuously verified as a “consensus scaffold” in various models.
2. Emerging growth points: Exosomes strengthen angiogenesis, hydrogels are used to regulate the immune microenvironment, and macrophage exosomes assist in bone repair. The past trend was relatively mild, but recently it has risen relatively fast (rising by more than 150% in 3 years), which indicates that the frontier research directions focus on vascularization, immune regulation, and microenvironment reconstruction.

The results show that the research has shifted from material structure repair to biological integrated functional regeneration. The continuous hotspots make the exosome-hydrogel research stable and repeatable, and the new growth points determine the innovation frontiers and patent opportunities. They provide a priority framework for the academic and translational agendas of future exosome-hydrogel research.

## 4 Discussion

We applied systematic semantic modeling and dual-source knowledge mining to reconstruct the knowledge structure and translational dynamics of the exosome–hydrogel system in regenerative medicine, particularly for bone repair. Parallel analyses of academic and patent data revealed long-term trends, entity coevolution, and temporal gaps between scientific discovery and technological application. The field is gradually shifting from basic exploration to application-oriented innovation, demonstrating that semantic data analysis and knowledge graph extraction effectively quantify the linkage between scientific insight and institutionalized patent expression.

Over the past decade, academic publications and patents have grown in parallel, showing an “exosomes–translation co-development” trend after 2021 as exosomes emerged as key cell communicators in regenerative medicine. Academic studies emphasize mechanistic clarity around “Exosome–hydrogel–bone regeneration,” whereas patents extend toward therapeutic applications, combinations, and device design—illustrating the transition from semantic convergence in research to conceptual expansion in intellectual property. Entity-level analysis confirms MSC-derived exosomes as the dominant source, with increasing emphasis on immune-related cells and osteoimmune regulation since 2021. From a material perspective, GelMA and collagen remain the leading hydrogels, balancing engineering tunability and biomimicry, while HA-based systems enhance angiogenesis and bone repair. Together, these findings depict a maturing and integrative paradigm linking osteogenesis, angiogenesis, and immunoregulation in exosome-assisted regenerative biomaterials.

With regards to evaluations, osteogenic markers are still important, but the increasing addition of angiogenic and immunoregulatory indicators signals a shift towards multidimensional evaluation. Recent studies have revealed that angiogenesis and osteogenesis are coupled processes driven by special vascular subtypes and local signaling conditions, and engineered bones increasingly emphasize coordination. Furthermore, osteoimmunology studies have shown that immune cells affect bone repair process and recent studies on macrophage polarization also indicate precise coordination between inflammation, angiosis and ossification processes. These studies reflect a shift from “structural recovery” to “functional regeneration,” which is mediated through an “osteogenesis–angiogenesis–immunoregulation” paradigm.

Material–endpoint co-occurrence analysis further uncovered both structural inertia and innovation potential. The high-density cluster of GelMA/collagen–osteogenesis combinations reflects the field’s reliance on standardized experimental configurations, which ensures reproducibility but also reveals methodological path dependence. In contrast, low-density regions associated with immune regulation and angiogenesis indicate emerging interdisciplinary frontiers characterized by diversity and rapid growth. Recent reviews emphasize that exosome-loaded hydrogels are evolving from single-target systems to multifunctional regenerative platforms combining immunoregulation, vascularization, and tissue remodeling, underscoring the need for studies to shift from “single-endpoint verification” to “multi-endpoint integration”.

Material–endpoint co-occurrence also revealed structural inertia and promise. The high density GMA/collagen–osteogenesis cluster highlights the field’s emphasis on standardized experiments, which is reproducible, but also the methodology is path-dependent, while low density regions for immune control and angiogenesis indicate emerging interdisciplinary research regions characterized by diversity and rapid growth. Recent reviews indicate that exosome loaded hydrogels are evolving from single-target systems to multi-cell regenerative systems involving immunoregulation, vascularization and remodeling, suggesting the need to move from “single-endpoint verification” to “multi-endpoints integration”.

Relations which are high-value yet unproven such as “hydrogel encodes exosome,” “exosome promotes angiogenesis”, and “extremes immune microenvironment” open substantial innovation opportunities. These proposals have already been proved in the literature, but have not been consistently proven for patent claims showing evidence–application gaps. Reviews have been highly critical in highlighting the significant advantages of an exosome–hydrogels system in controlled release and local microenvironment modulation, but it is still a methodological challenge to translate these mechanistic results into patentable ones. This motivates our evidence-linking and strength-ranking framework here. A potential limitation of this study is the reliance on open-source datasets and the lack of manual validation of NLP outputs.

From the methodological point of view, the main contribution of this paper is in modeling the narrative knowledge as structured and computable evidence in semantic-level terms. By combining topic modeling, entity recognition, and relation extraction, topic mapping moves from descriptive understanding to structural interpretation that has already been done in science knowledge mapping. The integration of academic and patent sources allows comparison on the same semantic level, which might reveal coupling between the scientific contents and the technology translation that could inform prediction on translational opportunity identification (Chen, 2006; Blei, 2012; Aria and Cuccurullo, 2017; Marx and Fuegi, 2020).

In conclusion, this study developed a systematic semantic modeling and knowledge graph framework based on a dual-source corpus integrating academic publications and patent documents. The main contributions are as follows: (1) Framework development: A dual-source, data-driven analytical framework was established to quantitatively map the research structure and translational landscape of exosome–hydrogel systems in regenerative medicine. This framework integrates semantic modeling, entity–relation extraction, and evidence-based ranking to provide an interpretable and traceable knowledge structure. (2) Academic–patent temporal dynamics: The analysis revealed a strong overall correlation but a persistent time lag between scientific discovery and patent deployment, reflecting the translational delay from mechanistic exploration to technological implementation. (3) Identification of innovation opportunities: Several high-evidence yet underrepresented academic propositions—such as MSC-derived exosomes in GelMA/collagen hydrogels and exosome-enhanced angiogenesis—were identified as potential targets for future translational research and patent strategies.

This evidence-based mapping paradigm not only bridges academic knowledge and technological application but can also be extended to other bioengineering systems to accelerate bench-to-patent translation.

## Data Availability

The original contributions presented in the study are included in the article/supplementary material, further inquiries can be directed to the corresponding author.

## References

[B1] Aria M. Cuccurullo C. (2017). Bibliometrix: an R-tool for comprehensive science mapping analysis. J. Informetr. 11, 959–975. 10.1016/j.joi.2017.08.007

[B2] Blei D. M. (2012). Probabilistic topic models. Commun. ACM 55, 77–84. 10.1145/2133806.2133826

[B3] Chen C. (2006). CiteSpace II: detecting and visualizing emerging trends and transient patterns in scientific literature. J. Am. Soc. Inf. Sci. Technol. 57, 359–377. 10.1002/asi.20317

[B4] Chen S. Tang Y. Liu Y. Zhang P. Lv L. Zhang X. (2019). Exosomes derived from miR-375-overexpressing human adipose mesenchymal stem cells promote bone regeneration. Cell. Prolif. 52, e12669. 10.1111/cpr.12669 31380594 PMC6797519

[B5] Chen L. Yu C. Xiong Y. Chen K. Liu P. Panayi A. C. (2023). Multifunctional hydrogel enhances bone regeneration through sustained release of stromal cell-derived factor-1α and exosomes. Bioact. Mat. 25, 460–471. 10.1016/j.bioactmat.2022.07.030 37056272 PMC10087917

[B6] Chen J. Chen J. Chen J. Lu R. Liu Z. Zhang Y. (2025). Pretreated exosomes by electrical stimulation accelerate bone regeneration. Bioact. Mat. 51, 383–398. 10.1016/j.bioactmat.2025.04.019 40491687 PMC12148642

[B7] Deerwester S. Dumais S. T. Furnas G. W. Landauer T. K. Harshman R. (1990). Indexing by latent semantic analysis. J. Am. Soc. Inf. Sci. 41, 391–407. 10.1002/(SICI)1097-4571(199009)41:6<391::AID-ASI1>3.0.CO;2-9

[B8] Du J. Li P. Haunschild R. Sun Y. Tang X. (2020). Paper-patent citation linkages as early signs for predicting delayed recognized knowledge: macro and micro evidence. J. Informetr. 14, 101017. 10.1016/j.joi.2020.101017

[B9] Fan J. Lee C.-S. Kim S. Chen C. Aghaloo T. Lee M. (2020). Generation of small RNA-Modulated exosome mimetics for bone regeneration. ACS Nano 14, 11973–11984. 10.1021/acsnano.0c05122 32897692 PMC7530137

[B10] Isik M. Vargel I. Ozgur E. Cam S. B. Korkusuz P. Emregul E. (2023). Human periodontal ligament stem cells-derived exosomes-loaded hybrid hydrogel enhances the calvarial defect regeneration in middle-age rats. Mat. Today Commun. 36, 106869. 10.1016/j.mtcomm.2023.106869

[B11] Jaffe A. B. (2002). Patents,Citations, and innovations: a window on the knowledge economy. Cambridge,Mass: MIT Press.

[B12] Kim E. M. Lee G. M. Lee S. Kim S. Lee D. Yoon D. S. (2022). Effects of mechanical properties of gelatin methacryloyl hydrogels on encapsulated stem cell spheroids for 3D tissue engineering. Int. J. Biol. Macromol. 194, 903–913. 10.1016/j.ijbiomac.2021.11.145 34838857

[B13] Lee J. Yoon W. Kim S. Kim D. Kim S. So C. H. (2020). BioBERT: a pre-trained biomedical language representation model for biomedical text mining. Bioinformatics 36, 1234–1240. 10.1093/bioinformatics/btz682 31501885 PMC7703786

[B14] Li W. Liu Y. Zhang P. Tang Y. Zhou M. Jiang W. (2018). Tissue-engineered bone immobilized with human adipose stem cells-derived exosomes promotes bone regeneration. ACS Appl. Mat. Interfaces 10, 5240–5254. 10.1021/acsami.7b17620 29359912

[B15] Li X. Fang S. Wang S. Xie Y. Xia Y. Wang P. (2024a). Hypoxia preconditioning of adipose stem cell-derived exosomes loaded in gelatin methacryloyl (GelMA) promote type H angiogenesis and osteoporotic fracture repair. J. Nanobiotechnology 22, 112. 10.1186/s12951-024-02342-6 38491475 PMC10943905

[B16] Li X. Si Y. Liang J. Li M. Wang Z. Qin Y. (2024b). Enhancing bone regeneration and immunomodulation *via* gelatin methacryloyl hydrogel-encapsulated exosomes from osteogenic pre-differentiated mesenchymal stem cells. J. Colloid Interface Sci. 672, 179–199. 10.1016/j.jcis.2024.05.209 38838627

[B17] Liu F. Sun T. An Y. Ming L. Li Y. Zhou Z. (2023). The potential therapeutic role of extracellular vesicles in critical-size bone defects: spring of cell-free regenerative medicine is coming. Front. Bioeng. Biotechnol. 11, 1050916. 10.3389/fbioe.2023.1050916 36733961 PMC9887316

[B18] Loper E. Bird S. (2002). “NLTK: the natural language toolkit,” in Proceedings of the ACL-02Workshop on effective tools and methodologies for teaching natural LanguageProcessing and computational linguistics (Stroudsburg, PA: Association for Computational Linguistics), 63–70. 10.3115/1118108.1118117

[B19] Lu W. Zeng M. Liu W. Ma T. Fan X. Li H. (2023). Human urine-derived stem cell exosomes delivered *via* injectable GelMA templated hydrogel accelerate bone regeneration. Mat. Today Bio 19, 100569. 10.1016/j.mtbio.2023.100569 36846309 PMC9945756

[B20] Lu P. Ruan D. Huang M. Tian M. Zhu K. Gan Z. (2024). Harnessing the potential of hydrogels for advanced therapeutic applications: current achievements and future directions. Signal Transduct. Target. Ther. 9, 166. 10.1038/s41392-024-01852-x 38945949 PMC11214942

[B21] Luo Z.-W. Li F.-X.-Z. Liu Y.-W. Rao S.-S. Yin H. Huang J. (2019). Aptamer-functionalized exosomes from bone marrow stromal cells target bone to promote bone regeneration. Nanoscale 11, 20884–20892. 10.1039/C9NR02791B 31660556

[B22] Manning C. Surdeanu M. Bauer J. Finkel J. Bethard S. McClosky D. (2014). “The stanford CoreNLP naturallanguage processing toolkit,” in Proceedings of 52nd annual meeting of theAssociation for computational linguistics: system demonstrations (Baltimore, MD: Association for Computational Linguistics), 55–60. 10.3115/v1/P14-5010

[B23] Marx M. Fuegi A. (2020). Reliance on science: worldwide front-page patent citations to scientific articles. Strateg. Manag. J. 41, 1572–1594. 10.1002/smj.3145

[B24] Neumann M. King D. Beltagy I. andAmmar W. (2019). “ScispaCy: fast and robust models for biomedical naturallanguage processing,” in Proceedings of the 18th BioNLP workshop and SharedTask (Florence, Italy: Association for Computational Linguistics), 319–327. 10.18653/v1/W19-5034

[B25] Porter A. L. Cunningham S. W. (2004). Tech mining: exploiting new technologies for competitive advantage. Hoboken: Wiley.

[B26] Sun J. Li G. Wu S. Zou Y. Weng W. Gai T. (2023). Engineering preparation and sustained delivery of bone functional exosomes-laden biodegradable hydrogel for *in situ* bone regeneration. Compos. Part B Eng. 261, 110803. 10.1016/j.compositesb.2023.110803

[B27] Tseng Y.-H. Lin C.-J. Lin Y.-I. (2007). Text mining techniques for patent analysis. Inf. Process. Manag. 43, 1216–1247. 10.1016/j.ipm.2006.11.011

[B28] Van Eck N. J. Waltman L. (2010). Software survey: vosviewer, a computer program for bibliometric mapping. Scientometrics 84, 523–538. 10.1007/s11192-009-0146-3 20585380 PMC2883932

[B29] Wu T. Wang L. Jian C. Gao C. Liu Y. Fu Z. (2024). Regulatory T cell-derived exosome mediated macrophages polarization for osteogenic differentiation in fracture repair. J. Control. Release 369, 266–282. 10.1016/j.jconrel.2024.03.028 38508525

[B30] Yang Z. Li X. Gan X. Wei M. Wang C. Yang G. (2023). Hydrogel armed with Bmp2 mRNA-enriched exosomes enhances bone regeneration. J. Nanobiotechnology 21, 119. 10.1186/s12951-023-01871-w 37020301 PMC10075167

[B31] Yoon B. Park Y. (2004). A text-mining-based patent network: analytical tool for high-technology trend. J. High. Technol. Manag. Res. 15, 37–50. 10.1016/j.hitech.2003.09.003

[B32] Zhang G. Feng Y. Yu G. Liu L. Hao Y. (2017). Analyzing the time delay between scientific research and technology patents based on the citation distribution model. Scientometrics 111, 1287–1306. 10.1007/s11192-017-2357-3

[B33] Zhang M. Li Y. Feng T. Li R. Wang Z. Zhang L. (2022). Bone engineering scaffolds with exosomes: a promising strategy for bone defects repair. Front. Bioeng. Biotechnol. 10, 920378. 10.3389/fbioe.2022.920378 35782499 PMC9240482

[B34] Zhao Y. Gong Y. Liu X. He J. Zheng B. Liu Y. (2022). The experimental study of periodontal ligament stem cells derived exosomes with hydrogel accelerating bone regeneration on alveolar bone defect. Pharmaceutics 14, 2189. 10.3390/pharmaceutics14102189 36297624 PMC9611133

